# Supplementary material for: Are routinely collected clinical and sociodemographic characteristics associated with social functioning and activities of daily living in schizophrenia? A machine learning approach descriptive of a schizophrenia cohort
Source: PLoS One. 2026 Apr 16;21(4):e0347326. doi: 10.1371/journal.pone.0347326 (PMC13086338; doi:10.1371/journal.pone.0347326)
Supplement: S1 Table — (DOCX) [file pone.0347326.s001.docx]

**S1 Table. Performance of each basis learner and of the ensemble algorithm.**

|  | ****GLM**** | ****GLM****  ****- RF scr.**** | ****GLM Int - RF scr.**** | ****GLMNet**** | ****GLMNet - RF**** ****scr.**** | ****RF**** | ****RF****  ****- RF scr.**** | ****XGB**** | ****XGB****  ****- RF scr.**** | ****SL (test)**** |
| --- | --- | --- | --- | --- | --- | --- | --- | --- | --- | --- |
| **SAS: PER** | 0.19 (0.01) | 0.17 (0.01) | 0.26 (0.02) | 0.15 (0.01) | 0.16 (0.01) | 0.76 (0.08) | 0.55 (0.07) | 0.43 (0.23) | 0.31 (0.17) | 0.01 (0.00) |
| **SAS: BAS** | 0.37 (0.01) | 0.35 (0.01) | 0.45 (0.04) | 0.36 (0.01) | 0.34 (0.01) | 0.74 (0.08) | 0.70 (0.10) | 0.57 (0.17) | 0.51 (0.10) | 0.35 (0.01) |
| **SAS: FIN** | 0.28 (0.01) | 0.26 (0.00) | 0.35 (0.02) | 0.27 (0.01) | 0.26 (0.00) | 0.76 (0.11) | 0.62 (0.00) | 0.43 (0.17) | 0.44 (0.20) | 0.14 (0.01) |
| **SAS: COM** | 0.19 (0.01) | 0.17 (0.01) | 0.22 (0.02) | 0.17 (0.01) | 0.17 (0.01) | 0.78 (0.12) | 0.65 (0.08) | 0.42 (0.27) | 0.39 (0.21) | 0.13 (0.01) |
| **SAS: SOC** | 0.24 (0.01) | 0.21 (0.01) | 0.22 (0.01) | 0.22 (0.01) | 0.20 (0.01) | 0.69 (0.14) | 0.48 (0.01) | 0.38 (0.10) | 0.39 (0.11) | 0.16 (0.01) |

The performance of each base learner is shown on the training set. The performance of the SuperLearner ensemble is presented for the testing set. Performance metrics are presented as the mean (standard deviation) R2 across the 20 imputed datasets.

Legend. SAS, Social Autonomy Scale; dimensions: PER, personal care; BAS, basic activities of daily living; FIN, management of financial resources; COM, complex activities of daily living; SOC, social and affective relationships;

GLM, General Linear Model; GLM - RF scr., General Linear Model with Random Forest screening; GLM Int - RF scr., General Linear Model with Interactions and Random Forest screening; GLMNet, General Linear Model with regularization; GLMNet - RF scr., General Linear Model with regularization and Random Forest screening; RF, Random Forest; RF - RF scr., Random Forest with Random Forest screening; XGB, eXtreme Gradient Boosting; XGB - RF scr., eXtreme Gradient Boosting with Random Forest screening; SL ens (test), SuperLearner ensemble (testing set);

SD, standard deviation.
