# Supplementary material for: Are routinely collected clinical and sociodemographic characteristics associated with social functioning and activities of daily living in schizophrenia? A machine learning approach descriptive of a schizophrenia cohort
Source: PLoS One. 2026 Apr 16;21(4):e0347326. doi: 10.1371/journal.pone.0347326 (PMC13086338; doi:10.1371/journal.pone.0347326)
Supplement: S2 Table — (DOCX) [file pone.0347326.s002.docx]

**S2 Table. Contribution of each base learner to the SuperLearner ensemble.**

|  | ****Mean**** | ****GLM Int - RF scr.**** | ****GLM**** | ****GLM****  ****- RF scr.**** | ****GLMNet**** | ****GLMNet - RF scr.**** | ****RF**** | ****RF****  ****- RF scr.**** | ****XGB**** | ****XGB****  ****- RF scr.**** |
| --- | --- | --- | --- | --- | --- | --- | --- | --- | --- | --- |
| **SAS: PER** | 0.02 (0.04) | 0.12 (0.07) | 0.00 (0.00) | 0.21 (0.11) | 0.00 (0.00) | 0.12 (0.12) | 0.28 (0.15) | 0.05 (0.09) | 0.10 (0.12) | 0.10 (0.11) |
| **SAS: BAS** | 0.00 (0.00) | 0.03 (0.04) | 0.07 (0.04) | 0.01 (0.02) | 0.53 (0.08) | 0.00 (0.00) | 0.20 (0.15) | 0.09 (0.10) | 0.04 (0.06) | 0.04 (0.06) |
| **SAS: FIN** | 0.02 (0.02) | 0.23 (0.10) | 0.07 (0.06) | 0.03 (0.06) | 0.00 (0.00) | 0.11 (0.10) | 0.29 (0.11) | 0.06 (0.07) | 0.05 (0.07) | 0.13 (0.11) |
| **SAS: COM** | 0.00 (0.01) | 0.04 (0.05) | 0.00 (0.00) | 0.39 (0.06) | 0.33 (0.15) | 0.03 (0.09) | 0.17 (0.09) | 0.00 (0.00) | 0.03 (0.08) | 0.00 (0.01) |
| **SAS: SOC** | 0.00 (0.00) | 0.02 (0.03) | 0.00 (0.00) | 0.05 (0.09) | 0.00 (0.00) | 0.65 (0.19) | 0.04 (0.08) | 0.13 (0.12) | 0.01 (0.02) | 0.11 (0.09) |

The influence of each base learner is represented by a coefficient ranging from 0 (no influence) to 1 (indicating that the SuperLearner relies entirely on that base learner), and presented as the mean (standard deviation) coefficients across the 20 imputed datasets.

NB: The “Mean” basis learner predicts outcomes using the average value of the target variable across all observations and is used as a benchmark.

Legend. SAS, Social Autonomy Scale; dimensions: PER, personal care; BAS, basic activities of daily living; FIN, management of financial resources; COM, complex activities of daily living; SOC, social and affective relationships;

GLM, General Linear Model; GLM - RF scr., General Linear Model with Random Forest screening; GLM Int - RF scr., General Linear Model with Interactions and Random Forest screening; GLMNet, General Linear Model with regularization; GLMNet - RF scr., General Linear Model with regularization and Random Forest screening; RF, Random Forest; RF - RF scr., Random Forest with Random Forest screening; XGB, eXtreme Gradient Boosting; XGB - RF scr., eXtreme Gradient Boosting with Random Forest screening; SL ens (test), SuperLearner ensemble (testing set).
