# Supplementary material for: Are routinely collected clinical and sociodemographic characteristics associated with social functioning and activities of daily living in schizophrenia? A machine learning approach descriptive of a schizophrenia cohort
Source: PLoS One. 2026 Apr 16;21(4):e0347326. doi: 10.1371/journal.pone.0347326 (PMC13086338; doi:10.1371/journal.pone.0347326)
Supplement: S1 Fig — (DOCX) [file pone.0347326.s003.docx]

**S1 Fig. Mean absolute SHAP value for the four dimensions of the Social Autonomy Scale showing meaningful performance of our model.**


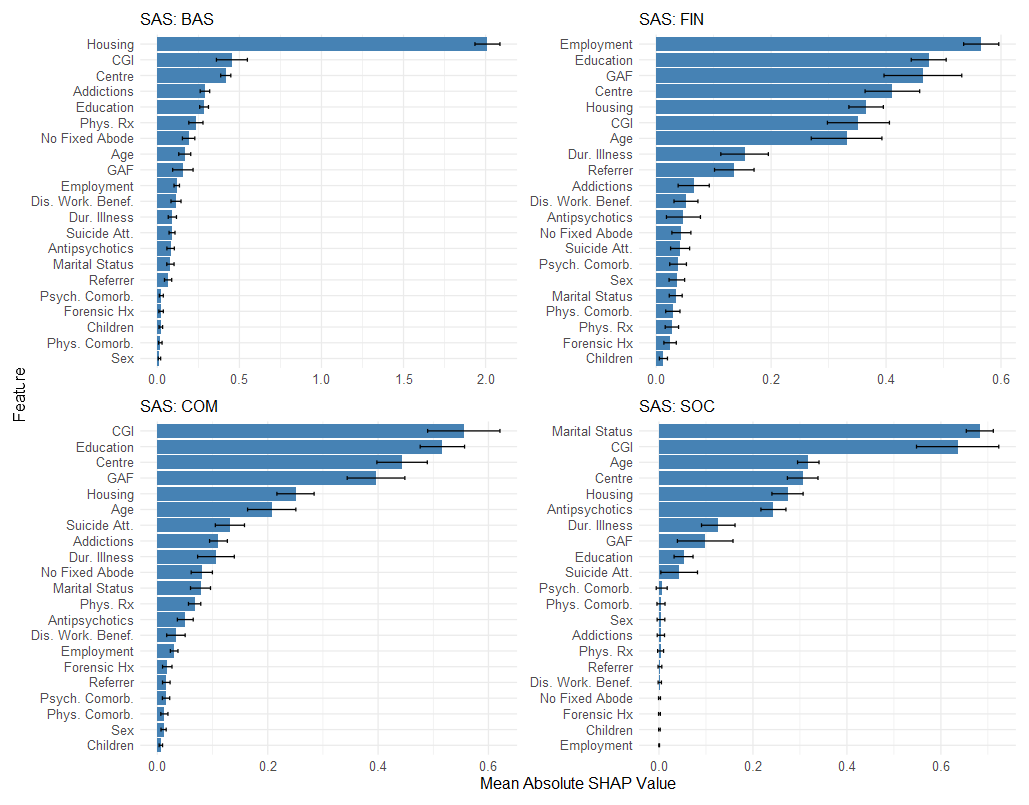


Averaged absolute SHAP values are calculated over the 20 imputed datasets and are represented on the x-axis. Predictors are represented on the y-axis. A higher averaged absolute SHAP value indicates a stronger absolute association with the outcome. Error bars represent ± 1 standard deviation (SD) from the mean.

Legend. SAS, Social Autonomy Scale; dimensions: BAS, basic activities of daily living; FIN, management of financial resources; COM, complex activities of daily living; SOC, social and affective relationships;

dipl., diploma; HS, High-School; Bach., Bachelor’s degree; Mast., Master’s degree; rel., relationship; Div./Wid., Divorced/Widowed; Gp H., Group Home; Fam. H., Family Home; Pers. H., Personal Home; Hless, Homeless; Empl., Employed; reg., regular; spec., specialized; Unempl., Unemployed; Dis Work. Benef., Disability Worker Beneficiary; FGA, first generation antipsychotic; SGA, second generation antipsychotic; Dur. Illness, Duration of illness; <2 yrs, less than 2 years;10 yrs+, 10 years or more; Psych., Psychiatric; Comorb., Comorbidities; Suicide att., Suicide attempts; Phys., Physical; 4+, 4 or more; Behav., Behavioral; Subst., Substance; Rx, Treatment; Hx, History; Pub. HC, Public HealthCare; Pr. HC, Private HealthCare; Soc. W., Social Worker; Pat., Patient.
