# Supplementary material for: Are routinely collected clinical and sociodemographic characteristics associated with social functioning and activities of daily living in schizophrenia? A machine learning approach descriptive of a schizophrenia cohort
Source: PLoS One. 2026 Apr 16;21(4):e0347326. doi: 10.1371/journal.pone.0347326 (PMC13086338; doi:10.1371/journal.pone.0347326)
Supplement: Attachment — Submitted filename: responses to reviewers.docx [file pone.0347326.s005.docx]

[*Reviewer #1: This manuscript addresses an important topic: whether routinely collected clinical and sociodemographic characteristics can predict social functioning and activities of daily living in schizophrenia using a machine learning approach.
The manuscript is clearly written, and the authors benefit from a large sample size and the use of advanced machine learning techniques. However, several issues require clarification before the manuscript can be considered for publication.*](https://journals.plos.org/plosone/s/file?id=ba62/PLOSOne_formatting_sample_title_authors_affiliations.pdf)

**Response. Many thanks for your positive feedback. Please see our detailed responses to your comments below. New text is highlighted in blue throughout the revised manuscript.**

*• Many variables known to predict functioning in schizophrenia (e.g., negative symptoms, cognitive performance, medication side effects) were not available. The authors should discuss how the absence of these variables may have affected model performance and interpretation.*

**Response. We have expanded our discussion regarding the limitations of missing variables (Discussion p.22).**

Second, although our set of socio-demographic and basic clinical predictors was extensive, it may still have omitted certain important factors. Notably, we did not include measures of negative symptoms, medication side effects, specific types of physical issues, or cognitive performance [43]. Additional factors such as insight into one’s disorder [44], psychiatric medications other than antipsychotics, and medication adherence may have also influenced functioning. We acknowledge that some of the variables routinely collected were not available in our database, and might have improved the predictive ability of our models. The omission of these clinical drivers not only reduces the model’s discriminatory power but also restricts the interpretation of our results. Specifically, our variable importance metrics (SHAP values) may be subject to residual confounding, which potentially masks the distinct contributions of our predictors to patient functioning.

*• The manuscript should clarify how the sample size was determined*

**Response. We have expanded the Methods section to clarify our sampling approach (p.6).**

Rather than relying on a predetermined sample size calculation, we employed an exhaustive inclusion strategy, utilizing all available observations from the database. This approach was chosen to maximize the statistical power of the analysis and to capture the full range of clinical variability present within the study population.

*• Clarify how categorical variables were encoded.*

**Response. We specified how categorical variables were encoded in the Methods section (p.7).**

Predictors (socio-demographic and clinical data)

The following background clinical and socio-demographic factors, as reported by clinical psychiatrists, were identified as potential predictors of functioning outcomes: center (six categories); age (continuous); sex (male vs. female); education (no high school diploma; high school diploma; Bachelor’s degree; Master’s degree); marital status (single; divorced/widowed; in a relationship); being a parent (yes vs. no); housing status (homeless; group home; family home; personal residence); employment (employed (regular); employed (specialized); unemployed); being a disabled worker beneficiary (yes vs. no); being of no fixed abode (currently; in the past; no); duration of illness (less than two years; two to five years; five to 10 years; more than 10 years); number of psychiatric admissions (nil; one; two; three; four; five to 10; more than 10); total time spent in hospital (nil; three months or less; three to six months; six to 12 months; more than 12 months); psychiatric comorbidities (yes vs. no); physical comorbidities (yes vs. no); addiction comorbidities (nil; behavioral only; substance only; both substance and behavioral); history of suicide attempts (no previous attempt; one; two; three; four or more); forensic history (yes vs. no); antipsychotic medication (nil; first-generation antipsychotics; second-generation antipsychotics; both); medication for physical disorders (yes vs. no); referrer (clinician from the public healthcare system; clinician from the private healthcare system; social worker; self-referral; other); severity score at the CGI (continuous); score at the GAF (continuous).

*• Specify whether the train/test split was stratified by center.*

**Response. We did not perform stratified splitting by center. Instead, we incorporated “center” as a categorical feature within the model. We have clarified this methodological decision in the revised manuscript (Methods, p. 9).**

We did not stratify the train-test split by center to avoid potential limitations in sample size for smaller centers. Instead, “center” was included as a categorical feature in our model to ensure the model could learn site-specific effects.

*• Some results require deeper discussion, such as:
• More suicide attempts being associated with better functioning.*

*• Psychiatric comorbidities being associated with better social relationships.*

**Response. We have provided a more detailed interpretation of these results in the revised manuscript (Discussion, p.21).**

We also observed unexpected relationships between a set of clinical factors and various dimensions of functioning. Individuals with a greater number of suicide attempts, those exhibiting psychiatric comorbidities or substance addiction demonstrated enhanced social functioning and/or ADL abilities. This may be due to a heightened access to support systems in individuals with more severe or complex disorders. This phenomenon, which can be interpreted through the lens of service engagement bias, suggests that patients presenting with more severe or high-risk clinical profiles are more frequently prioritized for intensive multidisciplinary care, vocational rehabilitation, and structured community support. While markers such as multiple suicide attempts or comorbid substance use indicate a profound clinical burden, they also serve as significant drivers for clinical entry into protected care pathways. Consequently, these individuals may receive a higher level of medical and social intervention compared to patients with milder symptomatic profiles, who may be less likely to trigger such rigorous support protocols and thus risk falling through the gaps of the care system. Our findings that individuals referred by professionals (compared to self-referrers) and beneficiaries of disability worker assistance exhibit better functioning further support this interpretation.

*Reviewer #2: The study focuses on schizophrenia; however, the current manuscript considers only behavioral and demographic measures, and the Introduction is insufficiently developed given the complexity of the disorder.*
**Response. Many thanks for your thorough review of our paper. Please see our responses to your comments below. New text is highlighted in blue throughout the revised manuscript.**

*Schizophrenia is a heterogeneous and stage-dependent disorder, with well-documented variability across disease phases and medication status. Contemporary neuroscience research increasingly aims to model the underlying neural mechanism disruptions and to translate them into quantitative biomarkers as following study:* [*https://doi.org/10.5455/apd.205512*](https://doi.org/10.5455/apd.205512)*,
In this context, the authors are encouraged to substantially expand the Introduction by discussing the full range of variables that characterize schizophrenia, with particular emphasis on studies investigating its neural signatures. It would also be appropriate for the authors to explicitly acknowledge that the current work adopts a limited perspective, focusing primarily on behavioral and demographic dimensions.*

**Response. We have expanded the second paragraph of our Introduction (p.4) to better situate our study within the broader context of contemporary neuroscience.**
Contemporary neuroscience models the neural dysfunctions associated with schizophrenia, translating them into quantitative biomarkers and behavioral phenotypes. Although these neural signatures are pivotal for elucidating the underlying pathophysiology of the condition, they remain absent from routine clinical assessment. From a clinical standpoint, research has shown that impairments in social functioning and ADLs among patients with schizophrenia are primarily related to several key factors, among which negative symptoms, such as loss of volition, withdrawal, inactivity, and muteness; and cognitive deficits in non-social (e.g. working memory, attention, executive functions) and social cognitive domains (e.g. emotional processing and mentalizing).

*In the Participants section, age-related details should be reported more rigorously. For example, the authors should provide mean age and standard deviation separately for women and men, rather than reporting aggregated statistics.*

**Response. We have added this information to the Results section (p. 11).**

Concordant with our previous studies [18,25], approximately three-quarters of the sample were male, with a mean age of 33 years (SD = 10; **Table 1**). Males had a mean age of 32.8 years (SD = 9.7), while females had a mean age of 34.0 years (SD = 11.0).

*In fact, in psychiatric disorders, medication status can substantially alter intrinsic brain dynamics, which in turn is expected to influence social behavior and related phenotypes as stated in following study:* [*https://dusunenadamdergisi.org/storage/upload/pdfs/1585638731-en.pdf*](https://dusunenadamdergisi.org/storage/upload/pdfs/1585638731-en.pdf)*. Consequently, heterogeneity in medication use among the modeled patient cohort may have a non-trivial impact on the reported results. The authors are therefore encouraged to systematically examine the dataset with respect to medication-related heterogeneity and to discuss its potential effects on both the predictive modeling outcomes and their interpretation.*
**Response. Thank you for this observation. We have improved our description of medication-related heterogeneity in the Results section (p.11).**

The majority of the cohort was prescribed second-generation antipsychotics, accounting for 600 individuals (63%). 72 patients (7.6%) were on first-generation antipsychotics alone, while patients prescribed both first- and second-generation represented 231 individuals (24%). A small minority (30 patients, 3.2%) were on no antipsychotic medication.

**We have addressed the effects of medication-related heterogeneity on predictive modeling outcomes and their interpretation in the Discussion (p.20).**

Medication-related heterogeneity contributed to predictive performance to a modest extent, though it was more pronounced for social and affective relationships. This is arguably due to clinical efficacy: comparatively to first generation antipsychotics, second generation antipsychotics may alleviate symptoms like emotional dysregulation and cognitive dysfunctions, enabling individuals to engage more effectively in social interactions and ADLs [40].

*The manuscript should clearly describe the clinical heterogeneity of the patient cohort. Relevant information includes: duration of illness (e.g., number of years since schizophrenia diagnosis), medication status (e.g., long-term medication use, newly initiated treatment, different medication classes), presence of comorbid conditions.
Explicitly reporting these factors is essential for interpreting the results and assessing their generalizability.*

**Response. We have rewritten our *Description of the Population* section to comply with the reviewer’s requirements (Results p.11).**

The majority of the cohort was prescribed second-generation antipsychotics, accounting for 600 individuals (63%). 72 patients (7.6%) were on first-generation antipsychotics alone, while patients prescribed both first- and second-generation represented 231 individuals (24%). A small minority (30 patients, 3.2%) were on no antipsychotic medication. The distribution of illness duration skewed toward chronic cases, with 414 individuals (44%) reporting a duration of 10 years or more. Patients with an illness duration between 5-10 years comprised 196 individuals (21%), followed by 153 patients (16%) in the 2-5 year range, and 125 patients (13%) with less than 2 years of illness. Regarding psychological comorbidities, the majority of the sample (693 patients, 73%) reported no such conditions, while 255 patients (27%) reported their presence. Physical comorbidity was present in 285 individuals (30%), with the remaining 657 (69%) reporting no physical comorbidities. 652 patients (69%) had no recorded suicide attempts, 143 individuals (15%) had one attempt, 54 (5.7%) had two, 24 (2.5%) had three, and 38 patients (4.0%) had four or more attempts. Additionally, a forensic history was absent in 774 patients (82%) and present in 145 patients (15%). Addiction patterns indicated that 385 individuals (41%) had no addictions, while 486 (51%) presented with substance-related addictions. Behavioral addictions were noted in 26 patients (2.7%), and 34 patients (3.6%) reported both substance and behavioral addictions. Finally, the majority of patients (759, 80%) were not currently receiving physical prescriptions, while 177 individuals (19%) were.

*The predictive models employed in the study are not sufficiently explained. The authors should clarify whether user-chosen or optimized parameters were used, whether patients were subgrouped or categorized, and how many participants were included in each group or category. In addition, it should be stated how many features were used to represent each individual.*
**Response. We have incorporated these details into the Analysis subsection of the Methods (p. 9).**

For each base learner, we used an "adaptive" hyperparameter tuning strategy [22] aiming to minimize logarithmic loss. Rather than an exhaustive, fixed grid search, this method dynamically manages the number of resampling iterations allocated to each hyperparameter combination. Briefly, the algorithm iteratively eliminates poorly performing parameter sets early in the process by employing an early-stopping mechanism based on model performance variance. This ensures that only the most robust parameter sets are evaluated across the full suite of resamples, significantly enhancing computational efficiency. For each outcome, data were split into training (70%) and hold-out testing (30%) sets. We did not stratify the train-test split by center to avoid potential limitations in sample size for smaller centers (Instead, “center” was included as a categorical feature in our model to ensure the model could learn site-specific effects). Base learners and the ensemble model were trained on the training set using 8-fold cross-validation (of note, using a greater number of folds led to computational issues). We trained the model on 21 features, consistent with the list provided in the *Predictors* section, excluding “number of psychiatric admissions” and “total duration of admission” due to their high FMI (see *Strategy for handling missing data*). Model performance was assessed on both the training set and the hold-out testing set for each of the 20 imputed datasets using the coefficient of determination (R2 metric).

*Given that the model appears to assume similarity among participants sharing certain characteristics, an important question remains unanswered: did the authors examine whether these individuals also exhibit similar neural brain dynamics or patterns? If not, do the authors plan to address this issue in a supportive and confirmatory future work framework?*
**Response. This suggestion has been incorporated in the Limitation section (Discussion, p.23).**

Seventh, it is important to acknowledge that the clinical heterogeneity observed in our study may partially stem from underlying variability in neurobiological processes. While our model is based on socio-demographic and clinical characteristics, it does not incorporate neural dynamics or brain imaging data, as these measures fall outside the scope of our behavioral and clinical focus. Future interdisciplinary investigations would be a logical next step to determine whether these behavioral groupings correspond to distinct neurobiological phenotypes.

*Please specify the software platform(s) on which the predictive models were implemented.*
**Response. We have updated the Methods section to explicitly describe our software environment (p.8).**

Data preprocessing and predictive modeling were performed in R (version 4.3.3).

*Finally, the manuscript does not address the stability and consistency of the proposed model. Were reliability analyses such as test–retest evaluations or intra-class correlation coefficients (ICC) conducted? If not, the authors should discuss this limitation. Without quantitative evidence of stability, it is difficult to assess the robustness of the proposed approach.*

**Response. In the Predictive Performance section, we mentioned that model performance remained stable across all imputed datasets, confirming the robustness of our results (Results, p.15).**

S1 Table shows the performance metrics across 20 imputed datasets for each of the five dimensions of the SAS. Averaged R2 for the SuperLearner ensemble on the hold-out testing sets was higher for basic ADLs (mean R2: 0.35, SD: 0.01) than for financial resources (0.14, 0.01), complex ADLs (0.13, 0.01), and social and affective relationships (0.16, 0.01). The predictive performance for personal care was very low (0.01, 0.00).

**We have updated Supplementary Figure 1 to illustrate the stability of mean absolute SHAP values across our imputed datasets.**

S1 Fig. Mean absolute SHAP value for the four dimensions of the Social Autonomy Scale showing meaningful performance of our model

Averaged absolute SHAP values are calculated over the 20 imputed datasets and are represented on the x-axis. Predictors are represented on the y-axis. A higher averaged absolute SHAP value indicates a stronger absolute association with the outcome. Error bars represent ± 1 standard deviation (SD) from the mean.

Legend. SAS, Social Autonomy Scale; dimensions: BAS, basic activities of daily living; FIN, management of financial resources; COM, complex activities of daily living; SOC, social and affective relationships;

dipl., diploma; HS, High-School; Bach., Bachelor’s degree; Mast., Master’s degree; rel., relationship; Div./Wid., Divorced/Widowed; Gp H., Group Home; Fam. H., Family Home; Pers. H., Personal Home; Hless, Homeless; Empl., Employed; reg., regular; spec., specialized; Unempl., Unemployed; Dis Work. Benef., Disability Worker Beneficiary; FGA, first generation antipsychotic; SGA, second generation antipsychotic; Dur. Illness, Duration of illness; <2 yrs, less than 2 years;10 yrs+, 10 years or more; Psych., Psychiatric; Comorb., Comorbidities; Suicide att., Suicide attempts; Phys., Physical; 4+, 4 or more; Behav., Behavioral; Subst., Substance; Rx, Treatment; Hx, History; Pub. HC, Public HealthCare; Pr. HC, Private HealthCare; Soc. W., Social Worker; Pat., Patient.

**Finally, the dispersion of SHAP values across the 20 imputed datasets was illustrated in Figures 1-4.**

*Reviewer #3: This manuscript presents a methodologically sound and ethically robust analysis of whether routinely collected clinical and sociodemographic variables can predict social functioning and activities of daily living in schizophrenia. Using a large multicenter dataset and appropriate machine-learning methods with independent validation, the authors provide a transparent and balanced assessment of predictive performance without overstating clinical utility. Limitations and counterintuitive findings are carefully acknowledged and interpreted cautiously, minimizing the risk of biased or misleading conclusions. Overall, the study makes a credible and incremental contribution.

** The data is not publicly accessible as the author mention about public data protection act, however the authors states that upon request it will be available.*

**Response. Many thanks for your positive feedback. We have revised our data availability statement in the new version of the manuscript.**

There are ethical and legal restrictions on sharing our de-identified data set publicly, as data contain potentially identifying or sensitive patient information (French National Advisory Committee for the Treatment of Information in Health Research, 16.060bis; French National Computing and Freedom Committee, DR-2017-268). Data requests may be sent to our internal committee (“Comite d’Acces aux Donnees de REHABase”, CAD-R) by direct contact with the corresponding author.

*Reviewer #4: The study addresses a clinically relevant question using a sophisticated machine learning pipeline and a well-characterized multicentric dataset. The manuscript is clearly written and methodologically ambitious. However, we have identified three areas-distinct from the limitations already acknowledged-that would substantially strengthen the scientific contribution and clinical applicability of this work.*

**Response. Many thanks for your feedback. Please see our responses to your comments below. New text is highlighted in blue throughout the revised manuscript.**

*(i) Inclusion of a Psychiatric or Normative Comparator Group
The current design describes predictors of functioning within schizophrenia, but cannot determine whether these predictors are disorder-specific or transdiagnostic. We recommend that the authors either: (a) include a control group of individuals with other severe mental illnesses (e.g., bipolar disorder, major depressive disorder) from the REHABase or a linked dataset, or (b) explicitly reframe the study scope to reflect that findings are descriptive of a schizophrenia cohort only. If the latter, this should be clearly stated in the title and abstract to avoid overgeneralization.*

**Response. We have revised our title and abstract to clarify that our analysis focuses exclusively on a cohort of patients with schizophrenia.**

**New version of the title:**

Are routinely collected clinical and sociodemographic characteristics associated with social functioning and activities of daily living in schizophrenia? A machine learning approach descriptive of a schizophrenia cohort.

**New version of the abstract:**

Patients with schizophrenia often experience substantial impairments in social functioning and activities of daily living (ADLs). Previous studies have highlighted links between sociodemographic and clinical factors and daily life functioning. However, routinely collected, non-scale-based clinical and sociodemographic characteristics have rarely been systematically evaluated as a standalone predictive framework. Understanding whether such variables alone can inform functional outcomes in schizophrenia may have important implications for public health. Using the French multicentric psychosocial rehabilitation database REHABase, we predicted five dimensions of the Social Autonomy Scale (a validated, clinician-administered questionnaire widely used in France): personal care, basic ADLs, financial autonomy, complex ADLs, and social and affective relationships. We used a SuperLearner ensemble machine learning method with a large set of routinely collected socio-demographic and basic clinical predictors descriptive of schizophrenia patients. Our sample comprised 948 participants. Averaged R2 on hold-out testing sets were higher for basic ADLs (mean R2: 0.35) than for social and affective relationships (0.16), financial autonomy (0.14), complex ADLs (0.13), and personal care (0.01). Factors associated with improved functioning included: being in a relationship, higher education, lower Clinical Global Impression scores, higher Global Assessment of Functioning scores, living in personal housing, being employed, mid-range illness duration, a history of suicide attempts and psychiatric comorbidities. Our findings indicate an association between socio-demographic and standard clinical variables routinely assessed in practice and outcomes in social functioning and ADLs. However, these variables account for only a limited proportion of the observed variance. This underscores the need for more specialized and precise assessments, e.g. based on cognitive abilities, to better understand and address patient functioning. We recommend targeted interventions focused on improving clinical symptoms, housing conditions, and supporting employment. Finally, clinicians should not assume that patients with seemingly protective factors, such as shorter illness duration, or absence of comorbidities, do not require further support.

*(ii) Temporal Validation to Support Prognostic Claims
The manuscript implies prognostic utility, yet the analysis is cross-sectional. We strongly encourage the authors to explore whether any follow-up data exist within REHABase (e.g., SAS reassessments at 6 or 12 months). Even a preliminary analysis of change scores or functional deterioration/progression would significantly enhance the paper’s translational value. If such data are not available, we recommend tempering claims about “predicting” outcomes in favor of “associating” or “explaining variance in.”*

**Response. We have revised the manuscript to explicitly acknowledge the associational nature of our findings.**

*(iii) Sensitivity and Stability Analysis for SHAP Interpretations
SHAP is a powerful interpretability tool, but its reliability is contingent on model and data stability. Given the variability in base learner weights and the risk of overfitting, we request that the authors report a measure of SHAP value stability (e.g., rank correlation of feature importance across imputed datasets, or 95% bootstrap intervals for mean absolute SHAP). This would allow readers to assess the confidence with which specific predictors (e.g., Master’s degree paradox can be translated into clinical heuristics.*

**Response. We have updated Supplementary Figure 1 to illustrate the stability of mean absolute SHAP values across our imputed datasets.**

S1 Fig. Mean absolute SHAP value for the four dimensions of the Social Autonomy Scale showing meaningful performance of our model.

Averaged absolute SHAP values are calculated over the 20 imputed datasets and are represented on the x-axis. Predictors are represented on the y-axis. A higher averaged absolute SHAP value indicates a stronger absolute association with the outcome. Error bars represent ± 1 standard deviation (SD) from the mean.

Legend. SAS, Social Autonomy Scale; dimensions: BAS, basic activities of daily living; FIN, management of financial resources; COM, complex activities of daily living; SOC, social and affective relationships;

dipl., diploma; HS, High-School; Bach., Bachelor’s degree; Mast., Master’s degree; rel., relationship; Div./Wid., Divorced/Widowed; Gp H., Group Home; Fam. H., Family Home; Pers. H., Personal Home; Hless, Homeless; Empl., Employed; reg., regular; spec., specialized; Unempl., Unemployed; Dis Work. Benef., Disability Worker Beneficiary; FGA, first generation antipsychotic; SGA, second generation antipsychotic; Dur. Illness, Duration of illness; <2 yrs, less than 2 years;10 yrs+, 10 years or more; Psych., Psychiatric; Comorb., Comorbidities; Suicide att., Suicide attempts; Phys., Physical; 4+, 4 or more; Behav., Behavioral; Subst., Substance; Rx, Treatment; Hx, History; Pub. HC, Public HealthCare; Pr. HC, Private HealthCare; Soc. W., Social Worker; Pat., Patient.

*Reviewer #5: This manuscript investigates whether routinely collected clinical and sociodemographic variables can predict social functioning and activities of daily living (ADLs) in individuals with schizophrenia using a SuperLearner machine-learning framework. The topic is clinically relevant and aligns with current efforts to integrate data-driven approaches into psychosocial rehabilitation research. The use of a large multicenter dataset (n=948) and ensemble modeling represents a notable methodological strength. Overall, the manuscript is clearly written and methodologically structured. However, several conceptual, methodological, and reporting issues require clarification before the manuscript can be considered for publication. In particular, concerns relate to model interpretation, generalizability, data availability compliance, and the clinical meaning of predictive performance.*
**Response. Many thanks. Please see our responses to your comments below.** **New text is highlighted in blue throughout the revised manuscript.**

*I provide detailed comments below.
1. Prediction vs. causation: The manuscript uses a predictive framework, but parts of the Discussion imply causal relationships. Please revise interpretations to avoid causal language.*

**Response. We have revised the manuscript to explicitly acknowledge the associational (rather than causal) nature of our findings.**

*2. Model performance and overfitting: Test-set performance appears modest with a noticeable gap between training and testing results. Provide clearer discussion on overfitting and the clinical relevance of the predictive accuracy.*

**Response. We have addressed the limitations regarding model overfitting in the Discussion section (p. 23).**

Sixth, performance metrics are derived exclusively from the testing set, which yielded lower predictive performance than the training set. This discrepancy provides clear evidence of overfitting, suggesting that the model captured idiosyncratic noise specific to the training data rather than exclusively learning generalizable clinical associations. In a clinical context, relying on training performance would be highly misleading, as it introduces an optimistic bias that overestimates the model's utility. Therefore, we emphasize testing set performance as the only trustworthy indicator of real-world applicability; despite the modest R2 values, this testing performance represents a realistic appraisal of how the model will perform when presented with new, unseen patients.

*3. Methodological transparency: Please clarify feature selection procedures, cross-validation design, and whether hyperparameter tuning was nested to avoid data leakage.*

**Response. We have provided more detailed methodological information in the Analysis section (Methods, p. 9) to clarify our approach and improve transparency.**

For each base learner, we used an "adaptive" hyperparameter tuning strategy [22] aiming to minimize logarithmic loss. Rather than an exhaustive, fixed grid search, this method dynamically manages the number of resampling iterations allocated to each hyperparameter combination. Briefly, the algorithm iteratively eliminates poorly performing parameter sets early in the process by employing an early-stopping mechanism based on model performance variance. This ensures that only the most robust parameter sets are evaluated across the full suite of resamples, significantly enhancing computational efficiency. For each outcome, data were split into training (70%) and hold-out testing (30%) sets. We did not stratify the train-test split by center to avoid potential limitations in sample size for smaller centers (Instead, “center” was included as a categorical feature in our model to ensure the model could learn site-specific effects). Base learners and the ensemble model were trained on the training set using 8-fold cross-validation (of note, using a greater number of folds led to computational issues). We trained the model on 21 features, consistent with the list provided in the *Predictors* section, excluding “number of psychiatric admissions” and “total duration of admission” due to their high FMI (see *Strategy for handling missing data*). Model performance was assessed on both the training set and the hold-out testing set for each of the 20 imputed datasets using the coefficient of determination (R2 metric).

*4. Missing data handling: Specify the imputation method, variables included in the imputation model, and how estimates were combined across imputed datasets.*

**Response. We have incorporated these details into the *Strategy for Handling Missing Data* section (Methods, p. 8)**

For each outcome measure, we removed observations with missing outcome values. However, some missing values remained in the predictors set. Overall, the percentage of missing data was of 2.8%. For individual variables, missing rates varied from 0% (for center, age, sex, psychiatric comorbidities) up to 14% for CGI and GAF, and 15% for total time spent in hospital. Our general strategy aimed to retain as many observations and variables as possible and impute missing values, ensuring the maintenance of a large number of observations and reducing the risk of selection bias. However, imputing missing data for “number of psychiatric admissions” and “total duration of admission” resulted in relatively unstable parameter estimates when applying standard general linear models during preliminary analyses (assessed by a fraction of missing information (FMI) > 0.40 [20]). After having excluded both these variables, parameter estimation did not fluctuate as much (FMI<0.10 except for CGI where FMI=0.19). We generated m=20 imputed datasets. Imputation was performed prior to predictive modeling and was conducted separately on the training and hold-out testing sets to prevent data leakage (see below). The imputation model incorporated the features described in the “Predictors” section, with the exception of “number of psychiatric admissions” and “total duration of admission”. We employed multiple imputation by chained equations (and the R package *mice*), using predictive mean matching for numeric variables, logistic regression for binary variables, and polytomous regression for unordered categorical variables. We derived R2 goodness-of-fit statistics and SHAP feature importance values (see below) independently for each imputed dataset. We then aggregated these results by calculating the mean and standard deviation across the 20 imputations.

*5. Interpretation of SHAP results: Emphasize that SHAP values reflect model behavior rather than causal importance of predictors.*

**Response. We have expanded the Limitations section (Discussion, p. 23) to explicitly acknowledge the limitations regarding SHAP values interpretation.**

It is important to emphasize that our study was not designed to establish causality; rather, our aim was to assess the predictive value of variables commonly collected in clinical practice. In particular, we emphasize that SHAP values reflect our predictive model behavior rather than the inherent causal significance of the predictors.

*6. Data availability statement: Further clarification is needed to ensure compliance with PLOS ONE’s data sharing policy (e.g., controlled-access repository or clear access procedure).*

**Response. We have revised our data availability statement in the new version of the manuscript.**

There are ethical and legal restrictions on sharing our de-identified data set publicly, as data contain potentially identifying or sensitive patient information (French National Advisory Committee for the Treatment of Information in Health Research, 16.060bis; French National Computing and Freedom Committee, DR-2017-268). Data requests may be sent to our internal committee (“Comite d’Acces aux Donnees de REHABase”, CAD-R) by direct contact with the corresponding author.

*7. Generalizability: Expand discussion on limitations related to the Social Autonomy Scale and potential clinician-rating bias.*

**Response. We have expanded our discussion of the limitations associated with the social autonomy scale in the Discussion section (p. 22).**

First, our measures of social functioning and ADLs were based on clinician rather than patient assessments. Consequently, our findings may be susceptible to observer bias.

Third, the social autonomy scale we used is primarily utilized and validated in France, which may limit the generalizability of our findings. While the original validation dates back to 1998, we are currently re-evaluating the instrument’s psychometric properties using contemporary datasets and advanced analytical techniques.
